# Optimizing Negotiation Conflict in the Cloud Service Negotiation Framework Using Probabilistic Decision Making Model

**DOI:** 10.1155/2015/858975

**Published:** 2015-10-12

**Authors:** Rajkumar Rajavel, Mala Thangarathinam

**Affiliations:** Department of Information Science and Technology, Faculty of Information and Communication Engineering, Anna University, Chennai, Tamil Nadu 600025, India

## Abstract

Optimization of negotiation conflict in the cloud service negotiation framework is identified as one of the major challenging issues. This negotiation conflict occurs during the bilateral negotiation process between the participants due to the misperception, aggressive behavior, and uncertain preferences and goals about their opponents. Existing research work focuses on the prerequest context of negotiation conflict optimization by grouping similar negotiation pairs using distance, binary, context-dependent, and fuzzy similarity approaches. For some extent, these approaches can maximize the success rate and minimize the communication overhead among the participants. To further optimize the success rate and communication overhead, the proposed research work introduces a novel probabilistic decision making model for optimizing the negotiation conflict in the long-term negotiation context. This decision model formulates the problem of managing different types of negotiation conflict that occurs during negotiation process as a multistage Markov decision problem. At each stage of negotiation process, the proposed decision model generates the heuristic decision based on the past negotiation state information without causing any break-off among the participants. In addition, this heuristic decision using the stochastic decision tree scenario can maximize the revenue among the participants available in the cloud service negotiation framework.

## 1. Introduction

Cloud resource provisioning offers high performance, availability, and throughput for their services after confirming the Service Level Agreement (SLA) with respect to predefined nonfunctional properties specifying Quality of Service (QoS) goals [1]. An SLA is defined as a contract made between the service consumer and the provider for promising their service access and provisioning mechanisms works according to the vision of cloud computing QoS goals [2]. The SLA types are broadly classified as static (provider predefined) SLA and negotiated (customized) SLA [3]. In general, cloud provider like Amazon and Microsoft Azure defines a common SLA for all the consumers that promises to guarantee 99.9% service availability. This is called provider predefined SLA which is automatically established and initiated after confirming the consumer service request with online payment. Sometimes, the cloud service provisioning mechanism in compliance to a set of predefined nonfunctional properties specified by the providers QoS goals may work as a semicustomized provisioning mechanism. In this case, the consumer with special QoS requirement cannot be satisfied by the current cloud provisioning mechanism due to its predefined SLA template. This limitation motivates the research focus towards the enforcement of automated negotiation framework in the cloud management system. It faces the challenges like (1) revenue maximization and providing differentiated SLA according to the types of clients [4] and (2) agreeing on personalized or customized service provisioning mechanism [5, 6].

Automated negotiation framework is identified as emerging research area for supporting the customized service provisioning in the SLA-oriented cloud management system [7, 8]. Optimizing the negotiation conflict among the participants (consumer, broker, and provider) available in such negotiation framework is a challenging issue for maximizing their revenue. A negotiation conflict is a disagreement among the participants which occurs due to egocentric misperception [9], aggressive (noncooperative) behavior [10], and uncertain preferences and uncertain goals [11] of their offers or counter offers generated during the bilateral negotiation process. This negotiation process consists of several negotiation stages (rounds) and it can be classified as initial, actual, and final rounds. Initial denotes the trial or first negotiation round, actual denotes the intermediate negotiation rounds, and final denotes the last negotiation round of the bilateral negotiation process. Therefore, the conflict that occurs during this bilateral negotiation process can be optimized according to the context of long-term and prerequest negotiation [12].

In prerequest context, the negotiation conflicts are identified during the trial round and get optimized before starting the actual negotiation process. This optimization is initiated by appropriately grouping the negotiation pairs having similar and nonaggressive behavior patterns. In order to perform the appropriate grouping, existing negotiation framework exploits the distance [13–15], binary [16, 17], context-dependent [18], and fuzzy similarity [19, 20] approaches. These kinds of existing similarity approaches will appropriately group the negotiators having similar negotiation preferences, which can optimize the success rate and communication overhead (number of interactions) among the negotiators. This optimization is guaranteed only during the trial negotiation round and not guaranteed to retain the optimization throughout the actual negotiation process. Because the behavioral patterns of the negotiators (negotiation pair) may not be constant in trial, actual, and final rounds of negotiation process, sometimes the negotiator behavioral patterns will change over the time interval depending on their interest and the perception about the opponent. Any misperception occurring at the time of negotiation process may lead to negotiation conflict. In certain cases, negotiators exhibiting contradictory or emotional behavioral patterns decided to ungroup from the similarity grouping process created during the trial negotiation round. In addition, the ungrouped negotiators are not encouraged to continue further negotiation process in the actual and final negotiation rounds because of its dissimilarity behavioral pattern.

The ungrouping decision made during the trail negotiation round may miss the opportunity of certain negotiators having the capability of reaching the negotiation outcome (agreement) because these negotiator behavioral patterns may generate the contradictory offer during the trial round and then gradually generate the complementary offer during the future actual and final rounds which has higher probability of reaching the agreement (success rate) with the opponent. Therefore, the optimization of negotiation conflict in the prerequest context (during the trial round) does not guarantee to provide higher success rate among the negotiators. So, this research work focuses on the optimization of negotiation conflict in the long-term context which can guarantee maximum success rate among the negotiators.

In case of long-term context, negotiation conflicts are identified and optimized during the entire sequence of exchanges that occur at each stage of the negotiation process. Further, the negotiation conflict is defined according to the types of conflict (dispute, crisis, limited violence, massive violence, abatement, and settlement) and transition occurs at each negotiation state [21]. These kinds of conflict occurring at each stage of negotiation process can be optimized through the appropriate behavioral patterns like collaborative, competitive, compromise, accommodating, and avoiding styles [22], followed by the corresponding negotiators decision making process. The proposed research work formulates the probabilistic decision making heuristic at each stage of negotiation process as the multistage Markov decision problem. In order to solve this problem, a novel probabilistic decision making approach is proposed using the stochastic decision tree scenario for optimizing the success rate and communication overhead among the negotiating participants available in the cloud service negotiation framework.

## 2. Cloud Service Negotiation Framework

**Figure 1:**
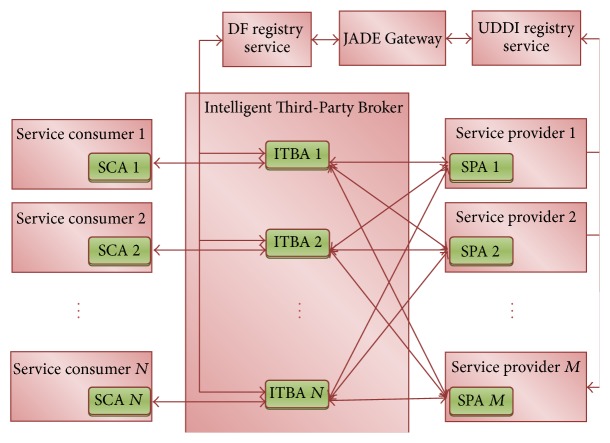
Architecture of cloud service negotiation framework.

The architecture of cloud service negotiation framework is proposed in the SLA-oriented cloud management system as shown in Figure 1. This framework consists of several components like service consumer, Intelligent Third-Party Broker, service provider, Universal Description Discovery and Integration (UDDI) registry, JADE Gateway agent, and Directory Facilitator (DF) registry. In order to automate the negotiation process among the participants (service consumers, Intelligent Third-Party Broker, and service providers), the agents such as service provider agents (SPAs), Intelligent Third-Party Broker Agents (ITBAs), and service consumer agents (SCAs) are introduced using the multiagent platform. These agents will mimic the behavior of service consumer, Intelligent Third-Party Broker, and service provider, respectively. Since the negotiation process automates through the agents, it supports only Agent Communication Language (ACL) message among the participants available in the multiagent platform. The cloud based SPAs will publish their available services in the UDDI registry using the Simple Object Access Protocol (SOAP) message. Therefore, the JADE Gateway agent is introduced as a language mediator for monitoring the web service description language modifications that occur in the UDDI registry. Also, it transparently updates the corresponding modifications in the DF registry for easily accessing the service using ACL message. So, the intermediate JADE Gateway agent exploits both SOAP and ACL messages for supporting the transparent communication between the UDDI and DF registries.

Initially, the SCAs request the ITBA to negotiate the service on its behalf, which chooses the best cloud service offer from the SPAs within a stipulated time. Then, the ITBA looks into the DF registry for choosing the appropriate SPA that matches the SCA request and starts the bilateral negotiation process with the concern SPA. Since ITBA negotiates on behalf of SCA, the actual negotiation process is modeled between the ITBA and SPA. The negotiation process exchanges the sequence of offer or counter offer between the broker agent *x*_*i*∈(1,*n*)_ and provider agent *y*_*j*∈(1,*m*)_ with respect to time period *T* ∈ {*T*_1_, *T*_2_,…, *T*_*n*_} pertaining to the fixed number of negotiation rounds NRs. At each negotiation round, broker agent *x*_*i*_ generates the offer or counter offer *O*_*x*_ based on the offer or counter offer *O*_*y*_ received from the provider agent *y*_*j*_. Then, the broker agent *x*_*i*∈(1,*n*)_ takes the appropriate decision to accept or reject the offer and sometime generates the counter offer based on the negotiation behavioral patterns suggested by its corresponding decision making heuristic. Acceptance of offer leads to negotiation commitment (agreement) and the rejection of counter offer decision leads to negotiation break-off or conflict between the participants. In order to optimize the negotiation conflict at each stage of negotiation process, the research problem is formulated as the Markov decision problem. Further, the negotiation conflict occurring at each negotiation stage is optimized using the proposed probabilistic decision making heuristic that is followed by the corresponding broker and provider agent's negotiation strategy.

## 3. Modeling the Broker and Provider Agent's Negotiation Strategy

The bilateral negotiation process exchanges the sequence of offer or counter offer between the broker agent *x*_*i*∈(1,*n*)_ and provider agent *y*_*j*∈(1,*m*)_ during the time period *T* ∈ {*T*_1_, *T*_2_,…, *T*_*n*_} as shown in the following equation:

(1) $$\begin{array}{c} {\text{offer}}_{x↔y}=\left\{\;O_{x}^{T_{1}},O_{y}^{T_{2}},O_{x}^{T_{3}},O_{y}^{T_{4}},\ldots ,O_{x}^{T_{n-1}},O_{y}^{T_{n}}\;\right\}. \end{array}$$

Let *O*_*x*_^*T*_1_^, *O*_*x*_^*T*_3_^,…, *O*_*x*_^*T*_*n*−1_^ and *O*_*y*_^*T*_2_^, *O*_*y*_^*T*_4_^,…, *O*_*y*_^*T*_*n*_^ denote the offers or counter offers generated by the broker and provider agents, respectively. The negotiation offer *O*_*x*_^*T*_1_^ consists of multiple negotiation attributes like price, time-slot, deadline, policy, and security level. These attributes change its values during the generation of offer or counter offer at each stage of negotiation process based on the negotiation behavioral patterns followed by the participating agent's negotiation strategy. The sequence of offer and counter offer exchanges during the negotiation process between the participants and is mapped into state-action form (cycle) as defined in the following equation:

(2) $$\begin{array}{c} {\text{offer}}_{x↔y}=\left\{\;s_{1},a_{1},s_{2},a_{2},\ldots ,s_{n-1},a_{n-1},s_{n},a_{n}\;\right\}. \end{array}$$

Let *s*_1_ and *a*_1_ represent the first negotiation state and its action at the time stamp *T*_1_. The action *a*_*k*∈(1,*n*)_ denotes the negotiator decision *d*_*k*∈(1,*n*)_ at time stamp *T*_*k*_ that can accept or reject the opponent offer and sometimes generate the counter offer response as defined in the following equation:

(3) akTkdkTk=resposex→yTk=acceptOyTk−1if  UOxTk≥UOyTk−1rejectOyTk−1if  UOxTk<UOyTk−1counterofferOxTkotherwise,

where *U*(*O*_*y*_^*T*_*k*−1_^) specify the utility value of offer received by the negotiator *x* and *U*(*O*_*x*_^*T*_*k*_^) corresponds to the utility value of counter offer response generated by the same negotiator with respect to the negotiation attributes. The additive utility function of the negotiator offer at any time stamp *T*_*k*_ can be computed under higher dimensional negotiation attribute as shown in the following equation:

(4) $$\begin{array}{c} U\left(\;O_{x\;\text{or}\;y}^{T_{k}}\;\right)=\overset{n}{\underset{i=1}{\sum }}w_{i}\times O_{xi}. \end{array}$$

Here, *O*_*x*1_, *O*_*x*2_,…, *O*_*xn*_ denotes the *n* number of negotiation attributes present in the offer *O*_*x*_ and *w*_*i*_ denote the weigh preference assigned for the respective negotiation attributes.

The negotiator decision employs different behavioral patterns for generating the counter offer response based on the utility value of their opponent offer received at each negotiation stage. Negotiator always expects high utility value (payoff) from the opponent's sequence of offers received during the negotiation process. Failing to receive such expected payoff at each negotiation stage leads to negotiation conflict between the negotiators. Such a negotiation conflict optimization is really difficult due to the lack of appropriate decision making heuristic at each negotiation stage that can increase the cooperation with the opponent negotiation behavioral patterns. Therefore, the negotiator decision making process at each negotiation stage is formulated as the multistage Markov decision problem that can generate the concessive counter offer response based on the heuristic decision suggested by the proposed probabilistic decision making model. This heuristic decision will maximize the success rate among the participants and thereby the revenue is maximized among the participants available in the cloud service negotiation framework.

### 3.1. Formulating Multistage Markov Decision Problem

**Figure 2:**
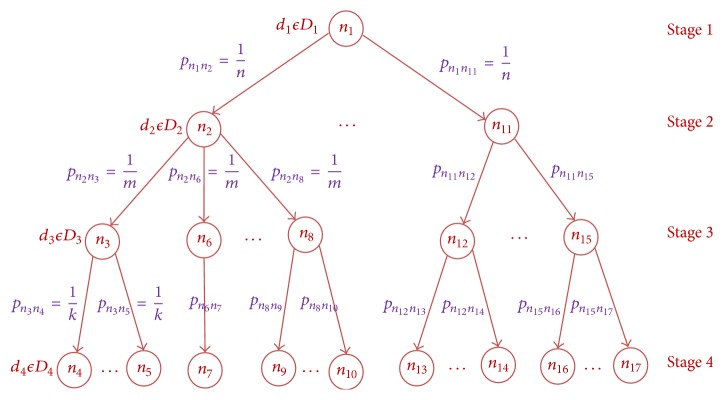
Probabilistic decision at each stage of negotiation process.

The probabilistic decision making model is developed for generating the heuristic decisions at each stage of the negotiation process by using the stochastic decision tree scenario as shown in Figure 2. A probabilistic decision making process based on the past negotiation state information formulates the multistage Markov decision problem on the probability space 〈*S*, *D*, *π*, *p*, *R*, *H*〉 as random parameters. Let *S* ∈ {*s*_1_, *s*_2_,…, *s*_*n*_} be the finite set of negotiation state spaces obtained during the finite negotiation process, *D* ∈ {*d*_1_, *d*_2_,…, *d*_*n*_} the finite set of decisions available at each state space, and *π* ∈ {*π*_1_, *π*_2_,…, *π*_*n*_} the finite set of negotiation policies (patterns) followed by the participants negotiation strategy. Any negotiation policy *π*_*t*_: *s*_*t*_ → *d*_*t*_ is the mapping that selects the appropriate decision *d*_*t*_ during the negotiation state *s*_*t*_. Then, *p* is the state transition function that defines the probability distribution as  *D* × *S* → *π*(*S*). The state transition probability *p*(*d*_*t*_, *s*_*t*_, *s*_*t*+1_) denotes the negotiation state transition from the current state *s*_*t*_ to the new state *s*_*t*+1_ based on the negotiator decision *d*_*t*_. Let *R* be the reward function denoted as *R*: *S* × *D* → *r*. Then, the reward function *R*(*s*_*t*_, *d*_*t*_) provides the reward value received by the broker agent, after confirming the decision *d*_*t*_ made in the negotiation state *s*_*t*_. Finally, *H* ∈ {*h*_0_^*t*_1_^, *h*_1_^*t*_2_^,…, *h*_*n*_^*t*_*n*_^} represents the set of all state transition history observed during the past negotiation states transitions at time instant *t* ∈ {*t*_1_, *t*_2_,…, *t*_*n*_}.

A negotiation process decision at each state is probabilistically modeled in terms of conditional probability distribution for receiving high probability of negotiation outcome without any conflicting result. At any time instant *t* ∈ *T*, the negotiation process present in any one of the negotiation states *S* ∈ {*s*_1_, *s*_2_,…, *s*_*n*_} makes the appropriate decision in the stochastic decision tree scenario for moving to the next state. Here, the decision term *d*_1_, *d*_2_,…, *d*_*k*_ represents the multinomial decision variables present in the nonterminals. The emergence of probabilistic decisions at the nonterminals is defined as follows:

(5) $$\begin{array}{c} p\left(\;\frac{d_{k}^{1}}{s_{x}},s_{x}^{0}\;\right),\\ p\left(\;\frac{d_{k}^{2}}{s_{x}},d_{k}^{1},s_{x}^{1}\;\right),\\ p\left(\;\frac{d_{k}^{3}}{s_{x}},d_{k}^{2},s_{x}^{2}\;\right),\\ \vdots \\ p\left(\;\frac{d_{k}^{T}}{s_{x}},d_{k}^{T-1},s_{x}^{T-1}\;\right). \end{array}$$

Let *d*_*k*_^1^, *d*_*k*_^2^,…*d*_*k*_^*T*^ denote the appropriate decision *d*_*x*_ ∈ *D* taken over the respective decision tree concerning the negotiation stage 1,2,…, *T*. Then, *s*_*x*_^0^, *s*_*x*_^1^,…, *s*_*x*_^*T*−1^ denotes the observed negotiation state of the respective negotiation stage 0,1, 2,…, *T* − 1. Similarly, the probabilistic decisions made in the terminals can be represented as shown in the following equation:

(6) $$\begin{array}{c} p\left(\;\frac{s_{y}}{s_{x}},d_{k}^{1},d_{k}^{2},d_{k}^{3},\ldots ,s_{x}^{T-1}\;\right). \end{array}$$

The broker agent (participant) negotiation strategy makes the choice of decision *d*_*k*_ ∈ *D* that actually depends on the current negotiation state *s*_*x*_. A transition probability from the negotiation states *s*_*x*_ to *s*_*y*_ at any time *t* ∈ *T* describes the set of state transition probabilities as defined in the following equation:

(7) $$\begin{array}{c} p_{s_{x}\to s_{y}}^{d_{k}}=\overset{n-1}{\underset{y=x+1}{\sum }}p_{s_{x}\to s_{y}}^{d_{k}},\;\forall s_{x}\in S,\;\;\forall d_{k}\in D. \end{array}$$

Assume that the summation of all state transition probability is equal to one; that is, [(*p*_*s*_*x*_→*s*_*x*+1__^*d*_*k*_^ + *p*_*s*_*x*+1_→*s*_*x*+2__^*d*_*k*_^ + ⋯+*p*_*s*_*x*+(*n*−2)_→*s*_*y*__^*d*_*k*_^) = 1] and ∀*s*_*x*_, ∀*s*_*y*_. The transition probability is 0 ≤ *p*_*s*_*x*_→*s*_*y*__ ≤ 1. The decision function *d*_*k*_ associated with the state transition from *s*_*x*_ to *s*_*y*_ receives the subsequent rewards as *r*_*s*_*x*_→*s*_*y*__^*d*_*k*_^. The objective of this probabilistic decision making problem is to find the sequence of appropriate decisions that maximizes the total expected reward *R*. It is depicted in the following equation:

(8) $$\begin{array}{c} \underset{k}{\mathrm{Max}}\left[\;R\left(\;s_{x},d_{k}\;\right)\;\right]. \end{array}$$

As revealed earlier, the value *r*_*s*_*x*_→*s*_*y*__^*d*_*k*_^ is the decision maker's (broker agent) reward obtained during the transition from state *s*_*x*_ to state *s*_*y*_ under the decision *d*_*k*_. The expected immediate reward function of this transition is defined as follows:

(9) $$\begin{array}{c} R\left(\;s_{x},d_{k}\;\right)=\overset{n-1}{\underset{x=1}{\sum }}\;\overset{n}{\underset{y=x+1}{\sum }}p_{s_{x}\to s_{y}}^{d_{k}}\times r_{s_{x}\to s_{y}}^{d_{k}}. \end{array}$$

Let *p*_*s*_*x*_→*s*_*y*__^*d*_*k*_^ be the state transition probability value obtained for the decision *d*_*k*_. The transition reward is formulated for computing the reward function in terms of computational values as shown in the following equation:

(10) rsx→sydk=1if  accept  rsx  before  transition  to  sy0.5if  counter  proposal  rsx  before  transition  to  sy0if  reject  rsx  before  transition  to  sy.

Assume that the broker agent negotiation strategy receives the reward values 1, 0.5, and 0 from the opponent denoting the acceptance, counter proposal, and rejection, respectively. The process of receiving high transition reward will optimize the negotiation conflict among the participants and thereby the negotiation outcome (success rate) and the participants' revenue are maximized in the cloud negotiation framework.

## 4. Experimental Evaluation

A novel probabilistic decision making model is implemented in the proposed cloud service negotiation framework. This decision making approach significantly optimizes the negotiation conflict during all the rounds of bilateral negotiation process. The validity of the proposed probabilistic decision making approach is verified through the comparison with the existing approaches available in the prerequest optimization context. The cloud based experimental testbed was created using Eucalyptus tool [23], for implementing the negotiation framework components on the virtual machines, that imitates the behavior of real cloud participants (consumer, broker, and provider). This cloud service negotiation framework testbed makes the bilateral negotiation process among the negotiating participants available in the real-time negotiation market possible. In order to automate the participants' negotiation process, a JADE tool [24] is incorporated over the testbed. In the cloud testbed, negotiating participants are configured according to the benchmark dataset shown in Table 1 [25, 26].

**Figure 3:**
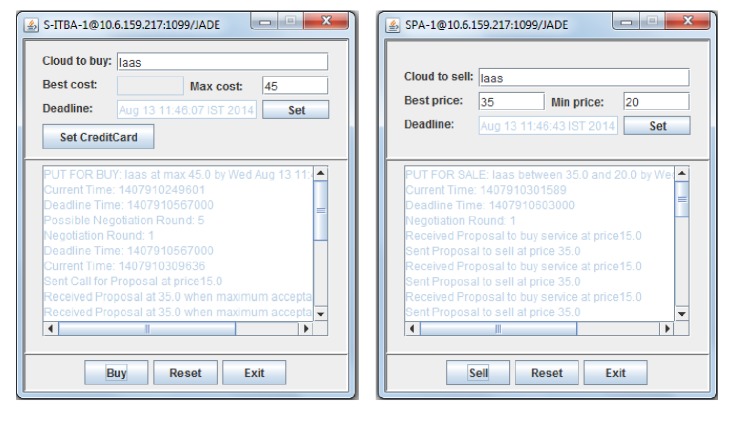
Preferences of broker and provider agent.

**Figure 4:**
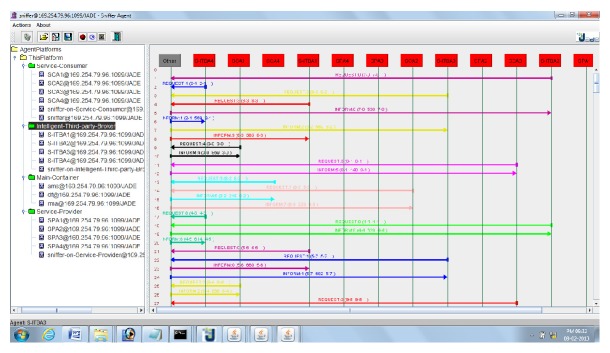
Visualization of negotiation process using sniffer agent.

In cloud testbed, start the bilateral negotiation process between the broker and provider agents by initializing their preferences as shown in Figure 3. Then, the corresponding sequence of offers and counter offers exchanged during the negotiation process are visualized through the sniffer agent as shown in Figure 4. The resulting performance of the proposed probabilistic decision approach is quantitatively measured against the existing distance, binary, context-dependent, and fuzzy similarity approaches with respect to different combination of negotiation pairs shown in Table 2. This quantitative measurement denotes the normalized values of the experimental results. In this connection, the distance of negotiation conflict optimality achieved by the proposed approach is measured in terms of success rate and communication overhead.

**Figure 5:**
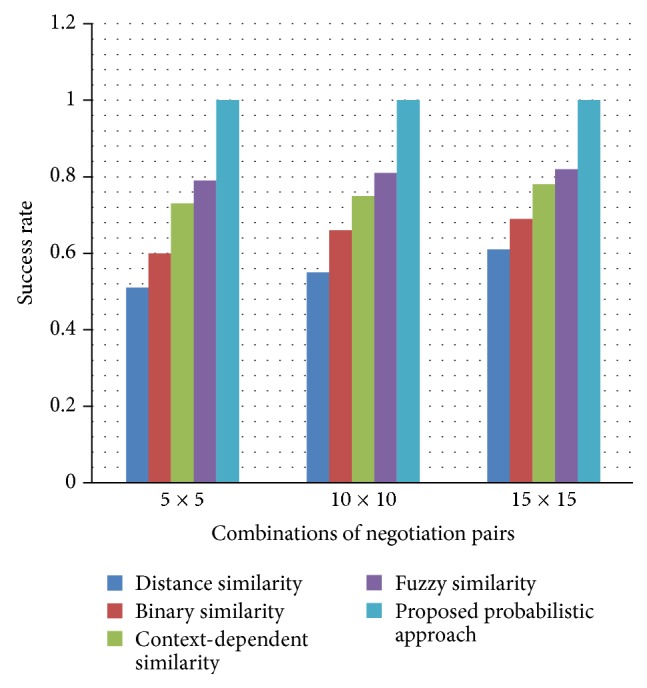
Achievement of success rate optimality.

**Figure 6:**
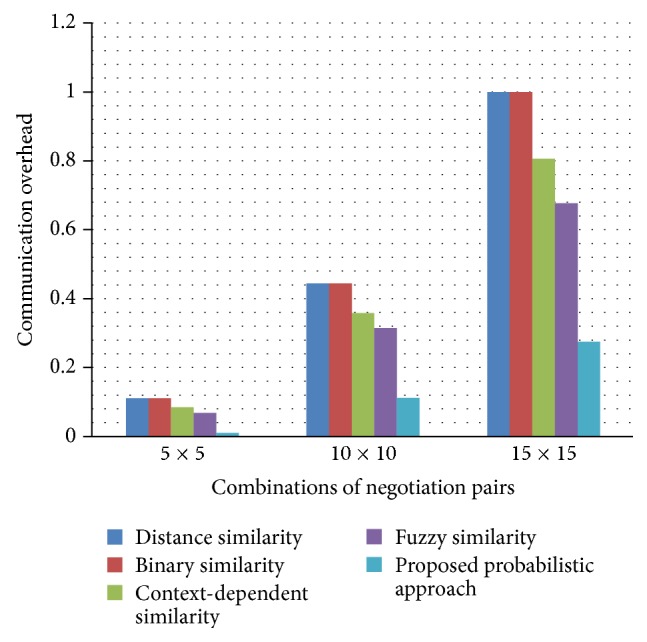
Achievement of communication overhead optimality.

The proposed probabilistic decision approach achieves 30% average success rate optimality over the existing approaches with respect to different combination of negotiation pairs as depicted in Figure 5. This significant improvement in the success rate is achieved due to the probabilistic decision making heuristic followed by the broker agent at each stage of negotiation process. Similarly, the communication overhead optimality shown in Figure 6 indicates that the broker agent using the probabilistic decision approach achieves 31.92% average improvement over the existing approaches. This significant improvement in the communication overhead occurs due to the earliest commitment of negotiation process made by the adaptive behavioral decision of the broker agent at each negotiation stage.

The proposed cloud service negotiation framework using the probabilistic decision approach can be integrated with the emerging MapReduce Framework for improving the parallel computing applications like bioinformatics, e-commerce, and distributed parallel machine scheduling. A reliable and efficient MapReduce Framework on the cloud computing technology can be employed for large-scale data processing and next-generation sequencing in the bioinformatics application [27]. In this way, the parallel processing can be introduced for optimal business in e-commerce application by assisting the intercommunication and negotiation process between the participants [28]. Moreover, an agent-based technology in the distributed parallel machine scheduling application needs to be improved with appropriate dynamic decision model in the preference of machines [29]. In order to support these future enhancements, the experimental data and related source code of the proposed cloud service negotiation framework are given in the appendix.

## 5. Conclusion and Future Work

The proposed cloud service negotiation framework includes the novel probabilistic decision making model for optimizing the negotiation conflict in long-term negotiation context. This heuristic decision generates appropriate counter offers at each stage of the negotiation process which maximizes the success rate among the negotiating participants. In addition, it minimizes the negotiation conflict through the appropriate behavioral decisions followed by the negotiator. An experimental evaluation revealed that the proposed probabilistic decision making model implemented in the cloud service negotiation framework achieves high success rate compared to the existing distance, binary, context-dependent, and fuzzy similarity approaches. This long-term context of negotiation conflict optimization can be applied to many real world application scenarios where the opponent's behavioral pattern is soft. Further, this research work can be enhanced using the hybrid combination of fuzzy and cognitive theory approach for effectively optimizing the negotiation conflict under uncertain behavioral information about the opponent. Applying a trust-based negotiation approach will also enhance the optimization of negotiation conflict among the participants.

## Figures and Tables

**Figure 7:**
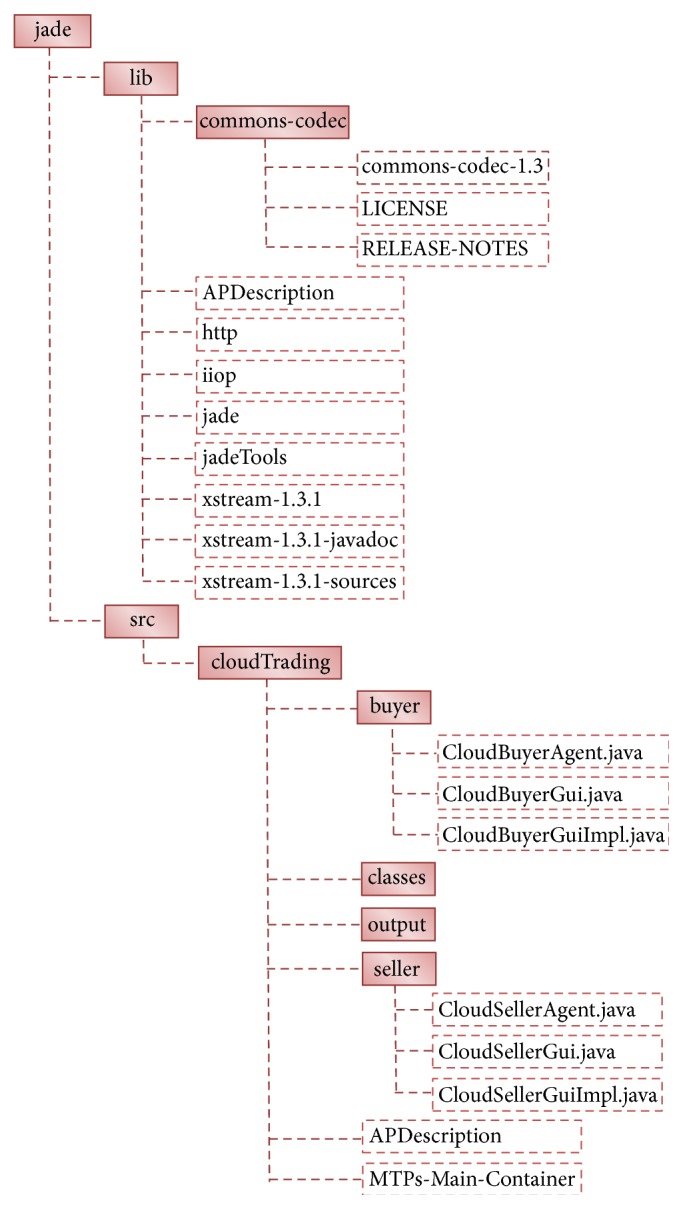
Source code package structure.

**Table 1:** Experimental settings of the negotiating participants.

Input parameters	Settings			
	Broker agent		Provider agent	
Initial price	[10,60]		[200,250]	
Reserved price	[200,250]		[10,60]	
Initial time-slot	[10,60]		[300,350]	
Reserved time-slot	[300,350]		[10,60]	
Negotiation deadline	[50,200] Rounds		[50,200] Rounds	
Negotiation agent	[5,20]		[5,20]	
Negotiation pattern	Conciliatory (0 < *π* < 1)	1/3	Conciliatory (0 < *π* < 1)	1/3
	Linear (*π* = 1)	1	Linear (*π* = 1)	1
	Conservative (1 < *π* < *∞*)	3	Conservative (1 < *π* < *∞*)	3

**Table 2:** Experimental results of different negotiation pairs of combination.

Similarity approach	Success rate			Communication overhead		
	5 × 5	10 × 10	15 × 15	5 × 5	10 × 10	15 × 15
Distance similarity	0.51	0.55	0.61	0.111	0.444	1.0
Binary similarity	0.60	0.66	0.69	0.111	0.444	1.0
Context-dependent similarity	0.73	0.75	0.78	0.085	0.358	0.806
Fuzzy similarity	0.79	0.81	0.82	0.068	0.315	0.677
Proposed probabilistic approach	1.0	1.0	1.0	0.010	0.112	0.275

**Table 3:** Experimental data source: cloud service negotiation framework.

Input data	Values	Settings	Operational role
Number of SCA	Integer	1–20	Subscribe service, negotiate through broker agent, accept, or reject the committed negotiation

Number of ITBA	Integer	1–20	Receive the negotiation request from consumer agent, follow multi-issue negotiation through the proposed negotiation strategy, and manage concurrent service negotiation with multiple service provider agents

Number of SPA	Integer	1–20	Publish service and follow multi-issue negotiation through various negotiation strategies, service provisioning

DF registry agent	Integer	1	Publish and discover agent services in the common broker-based cloud service negotiation market

UDDI registry agent	Integer	1	Publish and discover the services in the individual cloud service provider market

JADE Gateway agent	Integer	1	Monitor the WSDL modification in the UDDI and update the information in DF registry

## Appendix

### Experimental Data and Related Source Code

A series of business-to-business negotiation experiments are conducted for the cloud commerce application deployed over the cloud service negotiation framework, using the experimental data described in Table 3. To evaluate the performance of the multi-issue negotiation in the proposed cloud service negotiation framework, different decision making approaches are enforced in the cloud participants (service consumer agent, Intelligent Third-Party Broker Agent, and service provider agent) negotiation strategy.

The negotiation strategy tells the process: how the SCA, ITBA, and SPA negotiate among themselves using the standard protocol and algorithms. In the proposed ADSLANF, a bilateral negotiation is used between the SCA and TBA, and a multilateral negotiation (multiple bilateral negotiations) is used between the TBA and SPA. An alternate offer protocol is used for making communication among the parties which constitutes the negotiation operation. Initially, the offer is generated by the ITBA on behalf of SCA and the corresponding counter offer is generated by the opponent SPA during negotiation process. This process must be iterated until the negotiating parties come to an agreement. In this research work, a novel probabilistic decision making approach is used in the ITBA negotiation strategy for optimizing the negotiation conflict that occurs between the ITBA and SPA during their negotiation process. This heuristic decision in the ITBA generates appropriate counter offers at each stage of the negotiation process which maximizes the success rate among the negotiating participants. The implementation of actual negotiation process among the cloud participants is simulated in the JADE environment and the corresponding source code package structure is shown in Figure 7. In the package structure, the source code “CloudBuyerAgent.java” and “CloudSellerAgent.java” act as main parts of ITBA and SPA negotiation strategy for exchanging the sequence of offers and counter offers between the respective participants. The remaining executable source codes act as the supportive document for initiating the negotiation process in the cloud service negotiation framework. Since these source codes are having more than one thousand lines of codes in each class file, the authors have highlighted only the important functional statements present in the respective source codes (see Sourcecodes 1, 2, 3, 4, 5, and 6).

****Sourcecode 1: **CloudBuyerAgent.java.**
package cloudTradingNorConVsColCon.buyer;                        
import jade.core.^*∗*^;
import jade.core.behaviours.^*∗*^;
import jade.lang.acl.^*∗*^;
import jade.domain.^*∗*^;
import jade.domain.FIPAAgentManagement.^*∗*^;
import java.util.Vector;
import java.util.Date;
import java.util.^*∗*^;
import java.io.^*∗*^;
public class CloudBuyerAgent extends Agent
{
protected void setup() {
System.out.println("Buyer-agent "+getAID().getName()+" is ready.");
Object[] args = getArguments();
if (args != null && args.length > 0){
for (int i = 0; i < args.length; ++i) {
AID seller = new AID((String) args[i], AID.ISLOCALNAME);
sellerAgents.addElement(seller); }}
addBehaviour(new TickerBehaviour(this, 60000)
{
protected void onTick()
{
DFAgentDescription template = new DFAgentDescription();
ServiceDescription sd = new ServiceDescription();
sd.setType("Cloud-selling");
template.addServices(sd);
DFAgentDescription[] result = DFService.search(myAgent, template);
sellerAgents.clear();
for (int i = 0; i < result.length; ++i) {
sellerAgents.addElement(result[i].getName());
});}
protected void takeDown() {
if (myGui != null) {
myGui.dispose();}
System.out.println("Buyer-agent "+getAID().getName()+"terminated.");}
public void purchase(String title, float maxPrice, Date deadline) {
addBehaviour(new PurchaseManager(this, title, maxPrice, deadline));}
private class PurchaseManager extends TickerBehaviour
{
private PurchaseManager(Agent a, String t, float mp, Date d) {
super(a, 60000); // tick every minute
title = t;
maxPrice = mp;
deadline = d.getTime();
initTime = System.currentTimeMillis();
deltaT = deadline - initTime;}
public void onTick() {
long currentTime = System.currentTimeMillis();
if (currentTime > deadline) {
myGui.notifyUser("Deadline expired for buying "+title);
stop();}
else {
long elapsedTime = currentTime - initTime;
long negotiationTime = (deadline - initTime)/60000;
initialPrice=15;
if(negRound==0){
myGui.notifyUser("Current Time: "+initTime);
myGui.notifyUser("Deadline Time: "+deadline);
myGui.notifyUser("Possible Negotiation Round: "+negotiationTime);
float acceptablePrice = (float)Math.round(initialPrice);
myAgent.addBehaviour(new CloudNegotiator(title, acceptablePrice, this));
cPrice=acceptablePrice;}
else{
concessionPrice=concessionPrice+1;
float acceptablePrice = (float)Math.round(initialPrice+concessionPrice);
myAgent.addBehaviour(new CloudNegotiator(title, acceptablePrice, this));
cPrice=acceptablePrice;}
negRound+=1;
myGui.notifyUser("Negotiation Round: "+negRound);
myGui.notifyUser("Deadline Time: "+deadline);}}}
private class CloudNegotiator extends Behaviour
{
public CloudNegotiator(String t, float p, PurchaseManager m) {
super(null);
title = t;
maxPrice = p;
manager = m;}
public void action() {
switch (step) {
case 0:
myGui.notifyUser("Current Time: "+System.currentTimeMillis());
ACLMessage cfp = new ACLMessage(ACLMessage.CFP);
cfp.addReceiver((AID)sellerAgents.elementAt(i));
cfp.setContent(String.valueOf(cPrice));
cfp.setConversationId("cloud-trade");
cfp.setReplyWith("cfp"+System.currentTimeMillis()); // Unique value
myAgent.send(cfp);
myGui.notifyUser("Sent Call for Proposal at price"+cPrice);
mt = MessageTemplate.and(
MessageTemplate.MatchConversationId("cloud-trade"),
MessageTemplate.MatchInReplyTo(cfp.getReplyWith()));
case 1:
ACLMessage reply = myAgent.receive(mt);
BufferedWriter bw = new BufferedWriter(new FileWriter("C:/jade/src/project/output/ITBA5.txt", true));
bw.write(String.valueOf(reply.getSender().getLocalName()));
if (reply != null) {
if (reply.getPerformative() == ACLMessage.PROPOSE) {
pPrice=Float.valueOf(reply.getContent());
myGui.notifyUser("Received Proposal at "+pPrice+" when maximum acceptable price was "+cPrice);
bw.write(String.valueOf("PROPOSE"));
bw.write(String.valueOf(pPrice));
else if (pPrice == cPrice) {
ACLMessage orderAccept = new ACLMessage(ACLMessage.ACCEPT_PROPOSAL);
orderAccept.addReceiver(bestSeller);
orderAccept.setContent(String.valueOf(pPrice));
orderAccept.setConversationId("cloud-trade");
orderAccept.setReplyWith("order"+System.currentTimeMillis());
myAgent.send(orderAccept);
myGui.notifyUser("send Accept Proposal");
bw.write(String.valueOf("ACCEPT_PROPOSAL"));
bw.write(＇∖t＇);
bw.write(String.valueOf(pPrice));
step = 4; }}
else{
bw.write(String.valueOf("ACCEPT_PROPOSAL")); //}
else { block(); }
case 2:
BufferedWriter bw = new BufferedWriter(new FileWriter("C:/jade/src/project/output/ITBA5.txt", true));
if (bestSeller != null && bestPrice <= maxPrice) {
ACLMessage order = new ACLMessage(ACLMessage.ACCEPT_PROPOSAL);
order.addReceiver(bestSeller);
order.setContent(String.valueOf(pPrice));
order.setConversationId("cloud-trade");
order.setReplyWith("order"+System.currentTimeMillis());
myAgent.send(order);
myGui.notifyUser("send Accept Proposal");
mt = MessageTemplate.and(
MessageTemplate.MatchConversationId("cloud-trade"),
MessageTemplate.MatchInReplyTo(order.getReplyWith()));}
case 3:
BufferedWriter bw = new BufferedWriter(new FileWriter("C:/jade/src/project/output/ITBA5.txt", true));
reply = myAgent.receive(mt);
if (reply != null) {
Purchase order reply received
if (reply.getPerformative() == ACLMessage.ACCEPT_PROPOSAL) {
bw.write(String.valueOf("ACCEPT_PROPOSAL"));
bw.write(String.valueOf(pPrice));
ACLMessage commit = new ACLMessage(ACLMessage.ACCEPT_PROPOSAL);
commit.addReceiver(bestSeller);
commit.setContent(String.valueOf(pPrice));
commit.setConversationId("cloud-trade");
commit.setReplyWith("order"+System.currentTimeMillis());
myAgent.send(commit);
myGui.notifyUser("SLA Commit Proposal");
myGui.notifyUser("Cloud service successfully purchased.");}
}

****Sourcecode 2: **CloudBuyerGui.java.**
package cloudTrading.buyer;
public interface CloudBuyerGui
{
   void setAgent(CloudBuyerAgent a);
   void show();
   void hide();
   void notifyUser(String message);
   void dispose();
}

****Sourcecode 3: **CloudBuyerGuiImpl.java.**
package cloudTrading.buyer;
import jade.gui.TimeChooser;
import java.awt.^*∗*^;
import java.awt.event.^*∗*^;
import javax.swing.^*∗*^;
import javax.swing.border.^*∗*^;
import java.util.Date;
public class CloudBuyerGuiImpl extends JFrame implements CloudBuyerGui
{
public CloudBuyerGuiImpl()
{
super();
addWindowListener(new WindowAdapter() {
public void windowClosing(WindowEvent e) {
myAgent.doDelete();
}} );
setDeadlineB.addActionListener(new ActionListener(){
public void actionPerformed(ActionEvent e) {
Date d = deadline;
if (d == null) {
d = new Date();
}
TimeChooser tc = new TimeChooser(d);
if (tc.showEditTimeDlg(CloudBuyerGuiImpl.this) == TimeChooser.OK) {
deadline = tc.getDate();
deadlineTF.setText(deadline.toString());
}}} );
rootPanel.add(setCCB, gridBagConstraints);
buyB.addActionListener(new ActionListener(){
public void actionPerformed(ActionEvent e) {
String title = titleTF.getText();
if (title != null && title.length() > 0) {
if (deadline != null && deadline.getTime() > System.currentTimeMillis()) {
maxCost = Float.valueOf(maxCostTF.getText());
myAgent.purchase(title, maxCost, deadline);
notifyUser("PUT FOR BUY: "+title+" at max "+maxCost+" by "+deadline); }
catch (Exception ex1) {
JOptionPane.showMessageDialog(CloudBuyerGuiImpl.this, "Invalid max cost", "WARNING",
JOptionPane.WARNING_MESSAGE);
else {
JOptionPane.showMessageDialog(CloudBuyerGuiImpl.this, "Invalid deadline", "WARNING",
JOptionPane.WARNING_MESSAGE);}}
else {
JOptionPane.showMessageDialog(CloudBuyerGuiImpl.this, "No cloud title specified", "WARNING",
JOptionPane.WARNING_MESSAGE);
}}} );
resetB.addActionListener(new ActionListener(){
public void actionPerformed(ActionEvent e) {
titleTF.setText("");
desiredCostTF.setText("");
maxCostTF.setText("");
deadlineTF.setText(" ");
deadline = null;}} );
exitB.addActionListener(new ActionListener(){
public void actionPerformed(ActionEvent e) {
myAgent.doDelete();}} );
public void setAgent(CloudBuyerAgent a)
{
myAgent = a;
setTitle(myAgent.getName());
}
public void notifyUser(String message)
{ logTA.append(message+"∖n"); }                            
}

****Sourcecode 4: **CloudSellerAgent.java.**
package cloudTrading.seller;
import jade.core.Agent;
import jade.core.behaviours.^*∗*^;
import jade.lang.acl.ACLMessage;
import jade.lang.acl.MessageTemplate;
import jade.domain.^*∗*^;
import jade.domain.FIPAAgentManagement.^*∗*^;
import java.util.^*∗*^;
public class CloudSellerAgent extends Agent
{
protected void setup() {
System.out.println("Seller-agent "+getAID().getName()+" is ready.");
myGui = new CloudSellerGuiImpl();
myGui.setAgent(this);
myGui.show();
addBehaviour(new CallForOfferServer());
DFAgentDescription dfd = new DFAgentDescription();
dfd.setName(getAID());
ServiceDescription sd = new ServiceDescription();
sd.setType("Cloud-selling");
sd.setName(getLocalName()+"-cloud-selling");
dfd.addServices(sd);
DFService.register(this, dfd);}
protected void takeDown() {
if (myGui != null) {
myGui.dispose();}
System.out.println("Seller-agent "+getAID().getName()+"terminating.");
DFService.deregister(this);}
public void putForSale(String title, float initPrice, float minPrice, Date deadline) {
addBehaviour(new PriceManager(this, title, initPrice, minPrice, deadline));}
public class PriceManager extends TickerBehaviour {
private PriceManager(Agent a, String t, float ip, float mp, Date d) {
super(a, 60000); // tick every minute
title = t;
initPrice = ip;
currentPrice = initPrice;
deltaP = initPrice - mp;
deadline = d.getTime();
initTime = System.currentTimeMillis();
deltaT = ((deadline - initTime) > 0 ? (deadline - initTime): 60000);
bestPrice = initPrice;}
public void onStart() {
catalogue.put(title, this);
super.onStart();}
public void onTick()
{
long currentTime = System.currentTimeMillis();
if (currentTime > deadline) {
myGui.notifyUser("Cannot sell cloud"+title);
catalogue.remove(title);
stop();}}
public float getCurrentPrice() {
return currentPrice;}
private class CallForOfferServer extends CyclicBehaviour
{
private MessageTemplate mt = MessageTemplate.MatchPerformative(ACLMessage.CFP);
public void action() {
ACLMessage msg = myAgent.receive(mt);
if (msg != null) {
cPrice = Float.valueOf(msg.getContent());
myGui.notifyUser("Received Proposal to buy service at price"+cPrice);
ACLMessage reply = msg.createReply();
pPrice = pcurrentPrice;
if (cPrice != 0.0) {
if (msg.getPerformative() == ACLMessage.ACCEPT_PROPOSAL) {
ACLMessage commit = new ACLMessage(ACLMessage.ACCEPT_PROPOSAL); 
myAgent.send(commit);
myGui.notifyUser("SLA Commit at Price =" + bestPrice);
myGui.notifyUser("Cloud service successfully provisioned");}
else{if (cPrice < pPrice){
reply.setPerformative(ACLMessage.PROPOSE);
reply.setContent(String.valueOf(pPrice));}
else {
reply.setPerformative(ACLMessage.ACCEPT_PROPOSAL);
reply.setContent(String.valueOf(cPrice));
myGui.notifyUser("Accept Proposal at price"+ cPrice);}}}
else {
reply.setPerformative(ACLMessage.REFUSE);
myGui.notifyUser("Reject Proposal at price"+ cPrice);}
myAgent.send(reply);
myGui.notifyUser(cPrice != 0.0 ? "Sent Proposal to sell at price "+reply.getContent():  
"Refused Proposal as the cloud is not for sale");}
}

****Sourcecode 5: **CloudSellerGui.java.**
package cloudTrading.seller;
public interface CloudSellerGui
{
   void setAgent(CloudSellerAgent a);
   void show();
   void hide();
   void notifyUser(String message);
   void dispose();
}

****Sourcecode 6: **CloudSellerGuiImpl.java.**
package cloudTrading.seller;
import jade.gui.TimeChooser;
import java.awt.^*∗*^;
import java.awt.event.^*∗*^;
import javax.swing.^*∗*^;
import javax.swing.border.^*∗*^;
import java.util.Date;
public class CloudSellerGuiImpl extends JFrame implements CloudSellerGui
{
public void setAgent(CloudSellerAgent a) {
myAgent = a;
setTitle(myAgent.getName());}
public CloudSellerGuiImpl() {
super();
addWindowListener(new WindowAdapter() {
public void windowClosing(WindowEvent e) {
myAgent.doDelete();}} );
public void actionPerformed(ActionEvent e) {
Date d = deadline;
if (d == null) {
d = new Date();}
TimeChooser tc = new TimeChooser(d);
if (tc.showEditTimeDlg(CloudSellerGuiImpl.this) == TimeChooser.OK) {
deadline = tc.getDate();
deadlineTF.setText(deadline.toString());}}} );
gridBagConstraints = new GridBagConstraints();
gridBagConstraints.gridx = 3;
gridBagConstraints.gridy = 2;
gridBagConstraints.anchor = GridBagConstraints.NORTHWEST;
gridBagConstraints.insets = new Insets(5, 3, 0, 3);
rootPanel.add(setDeadlineB, gridBagConstraints);
rootPanel.setBorder(new BevelBorder(BevelBorder.LOWERED));
getContentPane().add(rootPanel, BorderLayout.NORTH);
logTA = new JTextArea();
logTA.setEnabled(false);
JScrollPane jsp = new JScrollPane(logTA);
jsp.setMinimumSize(new Dimension(300, 180));
jsp.setPreferredSize(new Dimension(300, 180));
JPanel p = new JPanel();
p.setBorder(new BevelBorder(BevelBorder.LOWERED));
p.add(jsp);
getContentPane().add(p, BorderLayout.CENTER);
p = new JPanel();
sellB = new JButton("Sell");
sellB.addActionListener(new ActionListener(){
public void actionPerformed(ActionEvent e) {
String title = titleTF.getText();
float desiredPrice = -1;
float minPrice = -1;
if (title != null && title.length() > 0) {
if (deadline != null && deadline.getTime() > System.currentTimeMillis()) {
desiredPrice = Float.parseFloat(desiredPriceTF.getText());
minPrice = Float.parseFloat(minPriceTF.getText());
if (minPrice <= desiredPrice) {
myAgent.putForSale(title, desiredPrice, minPrice, deadline);
notifyUser("PUT FOR SALE: "+title+" between "+desiredPrice+" and "+minPrice+" by "+deadline); }
else {
JOptionPane.showMessageDialog(CloudSellerGuiImpl.this, "Min price must be cheaper than best price", "WARNING",
JOptionPane.WARNING_MESSAGE);}}
else {
JOptionPane.showMessageDialog(CloudSellerGuiImpl.this, "Invalid deadline", "WARNING",
JOptionPane.WARNING_MESSAGE);}}
else {
JOptionPane.showMessageDialog(CloudSellerGuiImpl.this, "No cloud title specified", "WARNING",          
JOptionPane.WARNING_MESSAGE);}}} );
resetB = new JButton("Reset");
resetB.addActionListener(new ActionListener(){
public void actionPerformed(ActionEvent e) {
titleTF.setText("");
desiredPriceTF.setText("");
minPriceTF.setText("");
deadlineTF.setText("");
deadline = null;}} );
exitB = new JButton("Exit");
exitB.addActionListener(new ActionListener(){
public void actionPerformed(ActionEvent e) {
myAgent.doDelete();}} );
sellB.setPreferredSize(resetB.getPreferredSize());
exitB.setPreferredSize(resetB.getPreferredSize());
p.setBorder(new BevelBorder(BevelBorder.LOWERED));
getContentPane().add(p, BorderLayout.SOUTH);
setResizable(false);
}
public void notifyUser(String message) {
logTA.append(message+"∖n");}
}
